# Hydrogen sulfide limits neutrophil transmigration, inflammation, and oxidative burst in lipopolysaccharide-induced acute lung injury

**DOI:** 10.1038/s41598-018-33101-x

**Published:** 2018-10-02

**Authors:** Simone Faller, Florian Hausler, Andreas Goeft, Marc-Nicolas André von Itter, Veronica Gyllenram, Alexander Hoetzel, Sashko G. Spassov

**Affiliations:** Department of Anesthesiology and Critical Care Medicine, Medical Center – University of Freiburg, Faculty of Medicine, University of Freiburg, Freiburg, Germany

## Abstract

Transmigration and activation of neutrophils in the lung reflect key steps in the progression of acute lung injury (ALI). It is known that hydrogen sulfide (H_2_S) can limit neutrophil activation, but the respective mechanisms remain elusive. Here, we aimed to examine the underlying pathways in pulmonary inflammation. *In vivo*, C57BL/6N mice received the H_2_S slow releasing compound GYY4137 prior to lipopolysaccharide (LPS) inhalation. LPS challenge led to pulmonary injury, inflammation, and neutrophil transmigration that were inhibited in response to H_2_S pretreatment. Moreover, H_2_S reduced mRNA expression of macrophage inflammatory protein-2 (MIP-2) and its receptor in lung tissue, as well as the accumulation of MIP-2 and interleukin-1β in the alveolar space. *In vitro*, GYY4137 did not exert toxic effects on Hoxb8 neutrophils, but prevented their transmigration through an endothelial barrier in the presence and absence of MIP-2. In addition, the release of MIP-2 and reactive oxygen species from LPS-stimulated Hoxb8 neutrophils were directly inhibited by H_2_S. Taken together, we provide first evidence that H_2_S limits lung neutrophil sequestration upon LPS challenge. As proposed underlying mechanisms, H_2_S prevents neutrophil transmigration through the inflamed endothelium and directly inhibits pro-inflammatory as well as oxidative signalling in neutrophils. Subsequently, H_2_S pretreatment ameliorates LPS-induced ALI.

## Introduction

Acute lung injury (ALI) due to pulmonary inflammation still represents a major problem in critical care medicine and is associated with high rates of morbidity and mortality^1,2^. In this regard, postoperative pulmonary complications as underlying cause are of great importance^3,4^. By now, treatment or preventive options are limited, and new therapeutic strategies are needed.

ALI is characterised by alveolar barrier dysfunction, oedema formation, and accumulation of immune competent cells in the lungs. Especially the transmigration of neutrophils through endothelial cells promote the acute phase of pulmonary inflammation^5^. Activated neutrophils are attracted by pro-inflammatory cytokines, e.g., macrophage inhibitory protein-2 (MIP-2), to the side of the injury. Subsequently, neutrophils react with excessive pro-inflammatory cytokine release and oxidative burst, which in turn further aggravate the overall cellular inflammatory response and lung tissue injury^5^. Conversely, a reduction of neutrophil transmigration has been described to limit lung injury^6^.

In order to evaluate possible (pre)treatment options, we and others have previously shown that inhalation of hydrogen sulfide (H_2_S) prevents neutrophil accumulation in models of ventilator-^7–9^ and lipopolysaccharide (LPS)-induced lung injury^10–14^. Although recent data suggest that H_2_S may limit neutrophil activation and transmigration by downregulating pulmonary expression of chemoattractant molecules^15^ or by reducing leukocyte rolling and adhesion^15–18^, it remains completely unknown how H_2_S interacts with the neutrophilic inflammatory response.

The current study was designed to thoroughly investigate the impact of H_2_S on neutrophil vitality, transmigration, pro-inflammatory response, and oxidative burst *in vivo* and *in vitro*. We provide first evidence that H_2_S prevents neutrophil activation, migration, cytokine release, and oxidative burst upon LPS challenge, subsequently ameliorating lung tissue inflammation and injury.

## Results

### Effects of LPS and GYY4137 on acute lung injury and neutrophil transmigration *in vivo*

**Figure 1:**
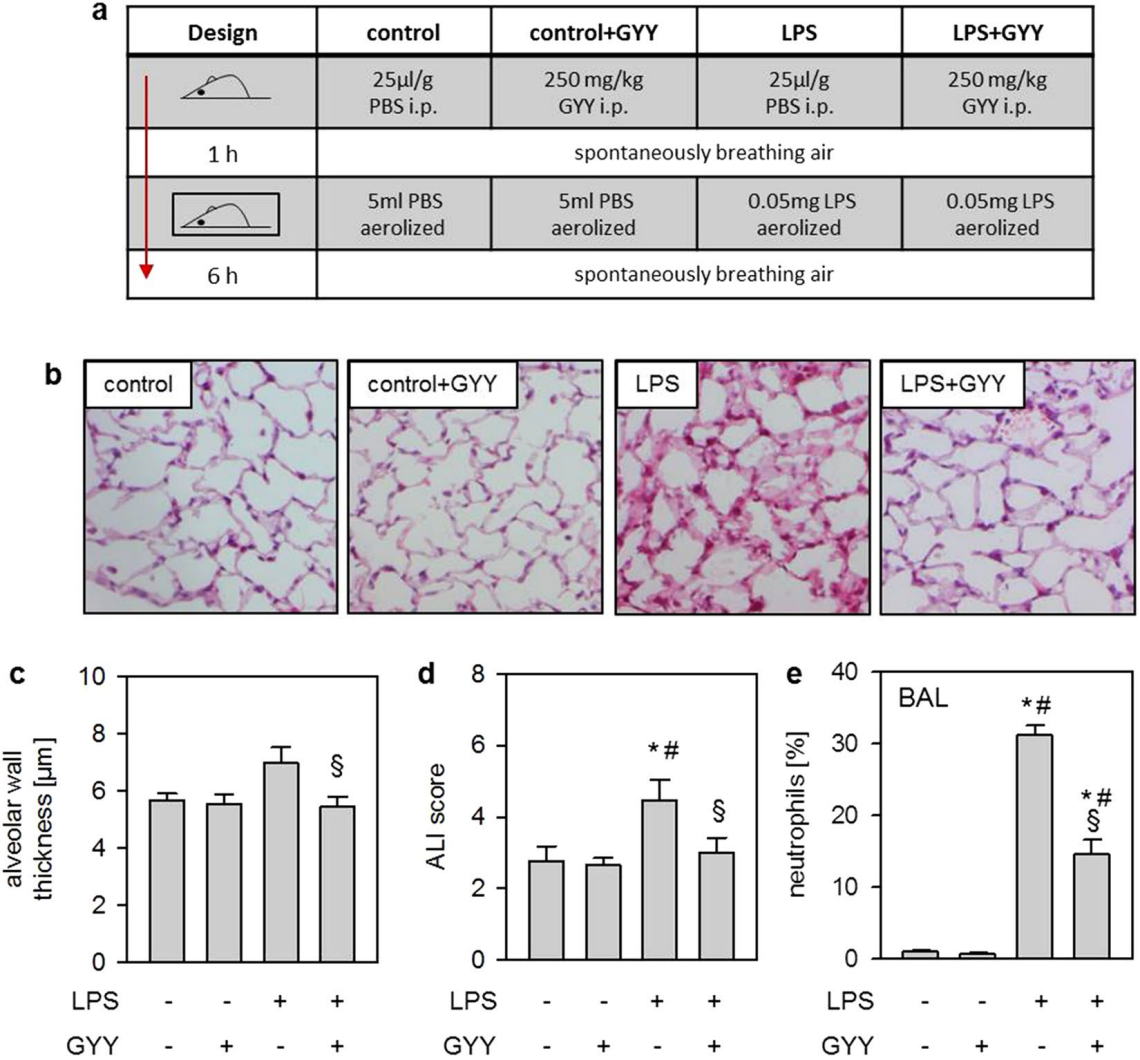
Effects of LPS and GYY4137 on acute lung injury and neutrophil transmigration *in vivo*. Control mice received 25 µl/g PBS i.p. and were nebulised 1 h later with 5 ml PBS. Control + GYY mice received 250 mg/kg GYY4137 i.p. and were nebulised 1 h later with 5 ml PBS (solved in PBS). The LPS group received 25 µl/g PBS i.p. and was nebulised 1 h later with 0.05 mg LPS (dissolved in 5 ml PBS), while the LPS + GYY group was treated with 250 mg/kg GYY4137 i.p. and was nebulised 1 h later with 0.05 mg LPS. All mice were euthanised after another 6 h (**a**). Sections from the left lung lobe were stained with haematoxylin and eosin. Representative pictures are shown for each experimental group (magnification = 200x; **b**). High power fields were randomly assigned to measure alveolar wall thickness (**c**) and to calculate an acute lung injury (ALI) score (**d**). The relative amount of neutrophils in the BAL fluid was determined by cytospin analysis (**e**). Data represent means ± SEM for n = 8/group. ANOVA (Tukey’s post hoc test), *LPS vs. control (**d**: P = 0.0076; **e**: P < 0.0001); *LPS + GYY vs. control (**e**: P < 0.0001); ^#^LPS vs. control + GYY (**d**: P = 0.0044; **e**: P < 0.0001); ^#^LPS + GYY vs. control + GYY (**e**: P < 0.0001); ^§^LPS + GYY vs. LPS (**c**: P = 0.0413; **e**: P < 0.0001).

First, we sought to induce acute lung injury in mice by nebulisation of LPS, either in the absence (LPS; study design Fig. 1a) or in the presence of the H_2_S releasing compound GYY4137 (LPS + GYY). Compared to controls (control) or controls receiving GYY4137 (control + GYY), treatment with LPS alone resulted in alveolar wall thickening after 6 h. In contrast, additional GYY4137 significantly decreased alveolar wall thickness (P = 0.0413; Fig. 1b,c). Similar results were obtained by determining an overall ALI score (Fig. 1d). The percentage of neutrophils in bronchoalveolar lavage (BAL) fluid was negligible in control and control + GYY groups. In contrast, neutrophil counts were elevated in the LPS group, while additional GYY4137 significantly reduced its number despite LPS treatment (LPS vs LPS + GYY P = 0.0088; Fig. 1e). These results indicate that GYY4137 mediates lung protection *in vivo* by limiting transmigration of neutrophils.

### Effects of LPS and GYY4137 on inflammatory response in the lung *in vivo*

**Figure 2:**
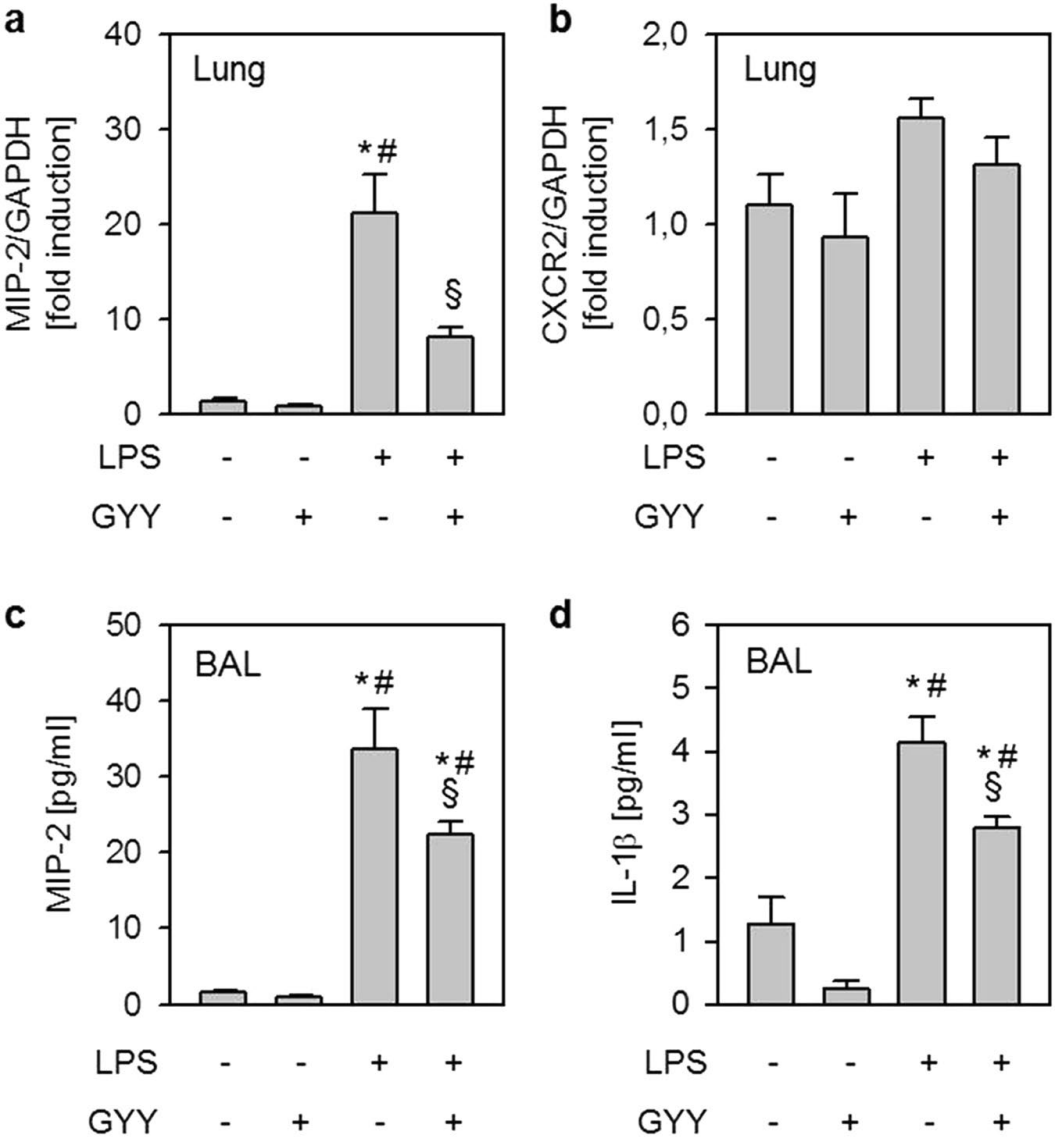
Effects of LPS and GYY4137 on inflammatory response in the lung *in vivo*. Control mice received 25 µl/g PBS i.p. and were nebulised 1 h later with 5 ml PBS. Control + GYY mice received 250 mg/kg GYY4137 i.p. and were nebulised 1 h later with 5 ml PBS (dissolved in PBS). The LPS group received 25 µl/g PBS i.p. and was nebulised 1 h later with 0.05 mg LPS (dissolved in 5 ml PBS), while the LPS + GYY group was treated with 250 mg/kg GYY4137 i.p. and was nebulised 1 h later with 0.05 mg LPS. All mice were euthanised after another 6 h. Lung homogenates were analysed for MIP-2 (**a**) and CXCR2 (**b**) and normalised to GAPDH by semi-quantitative polymerase chain reaction (a + b). The amount of MIP-2 (**c**) and IL-1β (**d**) in the BAL fluid was determined by ELISA. Graphs represent means ± SEM, n = 8/group. ANOVA (Tukey’s post hoc test), *LPS vs. control (**a**,**c**,**d**: P < 0.0001); *LPS + GYY vs. control (**c**: P = 0.0002; d: P = 0.0086); ^#^LPS vs. control + GYY (**a**,**c**,**d**: P < 0.0001); ^#^LPS + GYY vs. control + GYY (**a**,**c**,**d**: P < 0.0001) ^§^LPS vs. LPS + GYY (**a**: P = 0.0007; **c**: P = 0.0413; **d**: P = 0.0192).

Next, we analysed the expression of the chemotactic cytokine MIP-2 and its receptor C-X-C motif-chemokine receptor 2 (CXCR2) in lung tissue. Both are known mediators of neutrophil transmigration^5^. In contrast to control and control + GYY groups, LPS inhalation induced MIP-2 mRNA expression in lung tissue homogenates (LPS, Fig. 2a), an effect that was prevented by supplementary GYY4137 treatment (LPS + GYY, Fig. 2a). Analysis of CXCR2 mRNA expression yielded similar results, but failed statistical significance (Fig. 2b). In BAL fluid, the MIP-2 protein content remained minimal in both control groups (control, control + GYY, Fig. 2c). While LPS inhalation clearly increased MIP-2 protein (Fig. 2c), the additional application of GYY4137 significantly reduced the amount of MIP-2 as compared to LPS alone (P = 0.0413, Fig. 2c). Likewise, LPS inhalation increased the amount of interleukin-1β protein (IL-1β) in BAL fluid that was partially prevented in the presence of GYY4137 (P = 0.0192; Fig. 2d). Interesting to note, GYY4137 application per se tended to decrease IL-1β readings as compared to controls (Fig. 2d). According to these findings, the H_2_S releasing compound GYY4137 reduces neutrophil transmigration, most likely by limiting the accumulation of chemoattractant and pro-inflammatory cytokines in the lung.

### Effects of GYY4137 on Hoxb8 neutrophil vitality and migration through an endothelial monolayer *in vitro*

Different pulmonary cell type, e.g., endothelial or neutrophil cells, can produce MIP-2 and IL-1β upon LPS stimulation^5^. Because the results from our *in vivo* experiments would not allow to identify the specific cell type on which GYY4137 exerts its anti-inflammatory effects, we aimed to define the impacts of GYY4137 on differentiated Hoxb8 neutrophils^19^ *in vitro*.

**Figure 3:**
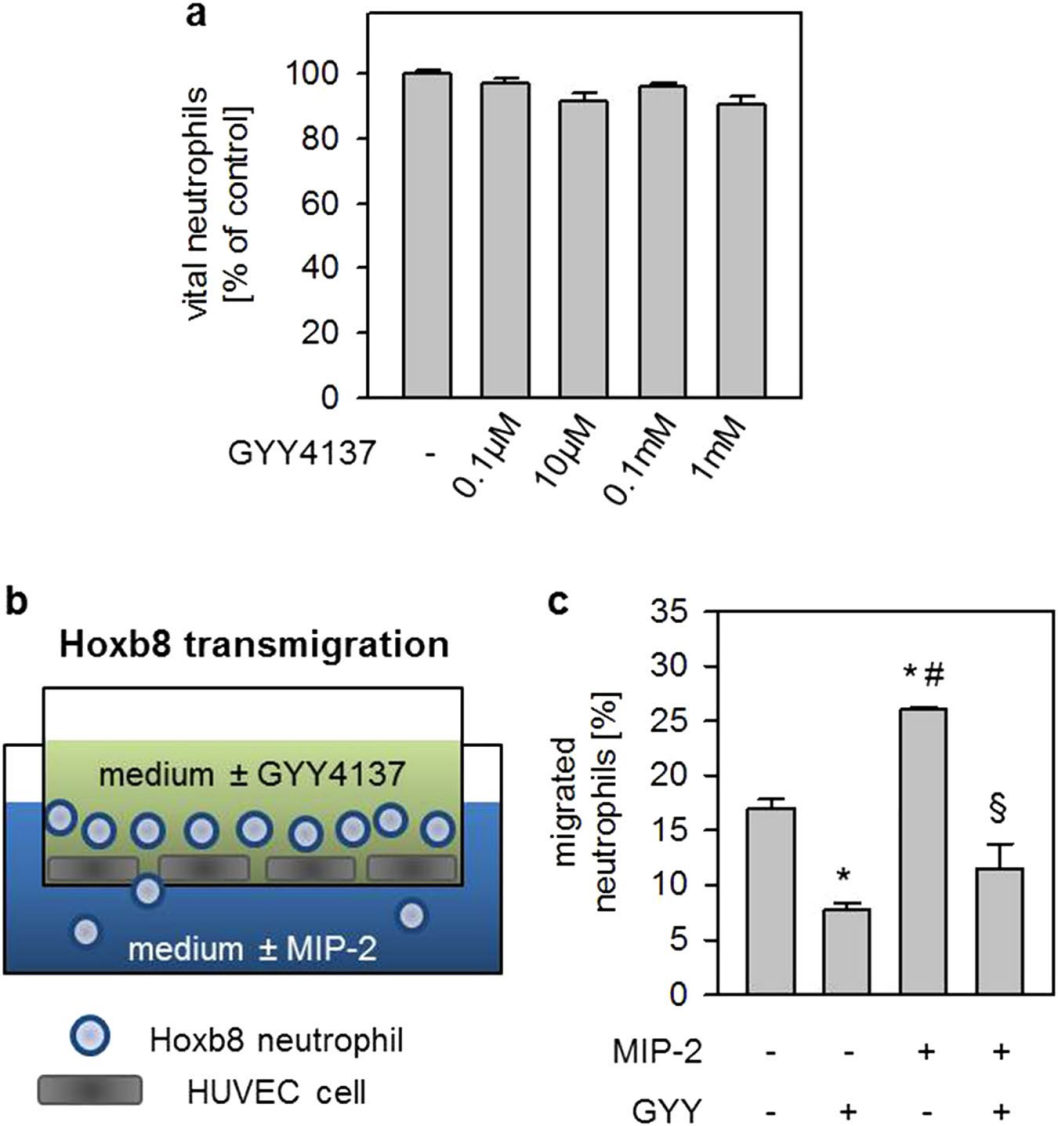
Effects of LPS or GYY4137 on Hoxb8 neutrophil vitality and migration through an endothelial monolayer *in vitro*. Differentiated neutrophils were incubated for 4 h in the absence (−) or presence (+) of GYY4137 as indicated, and the relative amount of vital cells was detected by propidium iodide staining and FACS analysis (**a**). Differentiated neutrophils were placed onto HUVEC endothelial monolayers in the upper compartment. Cell culture medium was either left untreated in all compartments (control) or was supplemented with 1 mM GYY4137 in the upper compartment (control + GYY). Cell culture medium in the lower compartment was supplemented with MIP-2, either in the absence (MIP-2) or presence of 1 mM GYY4137 in the upper compartment (MIP-2 + GYY, **b**). After incubation for 2 h, cells from the lower and upper compartment were stained separately with propidium iodide and analysed by FACS. The relative amount of migrated neutrophils was calculated as the ratio between vital cells in the lower compartment and the total number of vital cells (**c**). Graphs represent means ± SEM, n = 3/group. ANOVA (Tukey’s post hoc test), *GYY vs. control (**c**: P = 0.0030); *MIP-2 vs. control (**c**: P = 0.0039); ^#^MIP-2 vs. control + GYY (**c**: P < 0.0001); ^§^MIP-2 + GYY vs. MIP-2 (**c**: P = 0.0002).

We first investigated whether the inhibiting effect of GYY4137 on neutrophil accumulation might be a result of potential toxicity. To address this issue, Hoxb8 neutrophils were subjected to increasing concentrations of GYY4137. After 24 h of incubation, the cell vitality assays showed no differences between treated and untreated neutrophils (Fig. 3a).

After excluding potential toxicity, we further analysed whether GYY4137 directly affects neutrophil transmigration through an endothelial monolayer. As our results from co-culture experiments demonstrate (experimental design, Fig. 3b), MIP-2 led to an enhanced transmigration of neutrophils from the upper to the lower compartment (Fig. 3c). In contrast, incubation of neutrophils with GYY4137 in the upper compartment significantly reduced neutrophil transmigration through the endothelial monolayer (Fig. 3c). These effects have been observed in the absence of the stimulating cytokine (control vs control + GYY P = 0.0030; Fig. 3c) and were more pronounced in the presence of MIP-2 (MIP-2 vs MIP-2 + GYY P = 0.0002, Fig. 3c).

### Effects of LPS and GYY4137 on Hoxb8 neutrophil cytokine release and oxidative burst *in vitro*

**Figure 4:**
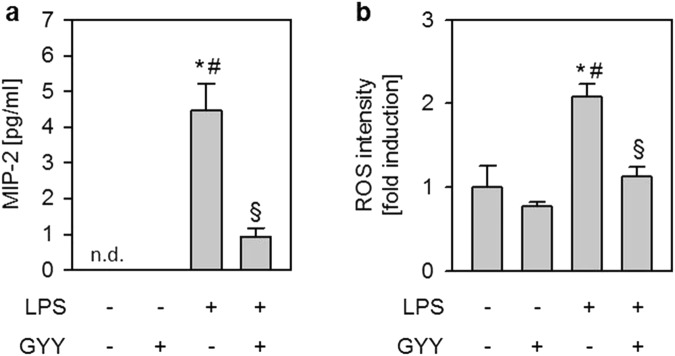
Effects of LPS and GYY4137 on Hoxb8 neutrophil cytokine release, oxidative burst and transmigration *in vitro*. Differentiated neutrophils were incubated for 4 h in the absence (control) or presence of 1 mM GYY4137 (control + GYY). In addition, neutrophils were treated with 100 ng/ml LPS in the absence (LPS) or presence of GYY4137 (LPS + GYY) as indicated. The amount of MIP-2 in cell culture supernatant was determined by ELISA (**a**). ROS intensity was detected by DHDHF-DA staining followed by FACS analysis (**b**). Graphs represent means ± SEM, n = 3/group. ANOVA (Tukey’s post hoc test), *LPS vs. control (**a**: P = 0.0002; **b**: P = 0.0062); ^#^LPS vs. control + GYY (**a**: P = 0.0002; **b**: P = 0.0019); ^§^LPS + GYY vs. LPS (**a**: P = 0.0010; **b**: P = 0.0130); n.d. not detectable.

Finally, we tested whether GYY4137 exerts direct anti-inflammatory and/or anti-oxidative effects on Hoxb8 neutrophils by analysing cytokine accumulation and reactive oxygen species (ROS) formation. LPS incubation profoundly induced MIP-2 accumulation in Hoxb8 neutrophils. In contrast, supplemental GYY4137 significantly reduced MIP-2 liberation despite LPS treatment (P = 0.0010; Fig. 4a). The formation of ROS was clearly induced by LPS compared to controls. Likewise, GYY4137 treatment in the presence of LPS completely prevented ROS production (P = 0.0130; Fig. 4b). The results from our *in vitro* experiments indicate that GYY4137 exerts direct inhibitory effects in neutrophil cells, thus preventing neutrophil migration, cytokine release, and ROS formation.

## Discussion

We and others have previously shown that hydrogen sulfide prevents lung injury in models of LPS-induced ALI^10,11,14^. In these studies, protection was clearly associated with the reduction of neutrophil accumulation in the lungs. Because in a series of injury models^7,8,10,13,20^, H_2_S-mediated inhibition of neutrophil influx appeared to be a major factor of its preventive effects, the rationale of the present study was to examine how H_2_S interferes with the neutrophilic inflammatory response. In order to specifically address the above mentioned problem of postoperative pulmonary complications as a source of ALI^3,4^, and to rule out potential preventive effects of H_2_S, we chose to apply the H_2_S releasing compound GYY4137 prior to the inflammatory insult.

We first induced ALI *in vivo* by nebulisation of LPS^21^. Lung injury was characterised by enlarged alveolar walls and an elevated ALI score. These findings are in line with the results of other rodent models, i.e., after intraperitoneal, intranasal, or intratracheal LPS application^10,12,13,22,23^. By contrast, prophylactic application of GYY4137, a water-soluble compound that slowly releases H_2_S^24^, prevented all signs of lung injury despite LPS treatment. Our data support the findings of two related studies. Here, GYY4137 exerted lung protection in a mouse endotoxemia model^12,13^. With regard to LPS-induced ALI, the observed effects of GYY4137 are comparable to those of H_2_S fast-releasing salts, i.e., sodium hydrosulfide^25^ or inhaled H_2_S gas^10,14^, underlining the lung protective properties of H_2_S irrespective of the form of application. Although we chose a ‘prophylactic’ time point of GYY4137 application, it seems likely that a more ‘therapeutic’ approach, i.e., administration after the injurious insult, can reduce lung inflammation and injury. First, GYY4137 showed anti-inflammatory effects in several experimental models using a post-injury time points of application^12,26^. Second, we recently demonstrated the time dependency of application^27^. After setting the insult, an earlier H_2_S inhalation resulted in a more protective effect. However, postponed application still reduced lung inflammation^27^. Mechanistically, it has been demonstrated in LPS and other models that H_2_S-mediated lung protection is associated with the reduction of inflammatory processes. Amongst them, H_2_S significantly decreases the activity of NF-κB^26,28–32^, cystathionine-β-synthetase and cystathionine-γ-lyase^14,33^, and particularly pro-inflammatory cytokine and neutrophil accumulation in the lungs^7–10,12,13,20,34^.

Pulmonary neutrophil activation and transmigration display key events in the development of ALI after bacterial challenge^5^. Here, we show that upon LPS inhalation a substantial fraction of neutrophils was recruited into the alveolar space that was inhibited in the presence of GYY4137. These results are in line with previous work on endotoxemia in rats and mice demonstrating a decrease in lung myeloperoxidase activity and neutrophil influx in response to GYY4137 application^11–13^. While the limitation of neutrophil function appears to be a general mechanism of H_2_S-mediated protection in various lung injury models^7–10,12,13^, the exact mechanism remains elusive. We therefore investigated the effect of the H_2_S releasing compound GYY4137 on neutrophil reaction upon LPS stimulation *in vivo* and *in vitro*.

MIP-2 represents a central chemotactic mediator that is released following LPS challenge in various pulmonary cell types, e.g., endothelial cells^35,36^, epithelial cells^37,38^, or neutrophils^5,10^, and subsequently activates the neutrophil inflammatory response. In the current study, we found that LPS increased MIP-2 mRNA expression in lung tissue homogenates and MIP-2 protein in the alveolar space. In contrast, GYY4137 application inhibited both MIP-2 mRNA expression and -protein release even in the presence of LPS. Furthermore, GYY4137 tended to reduce mRNA expression of the corresponding receptor CXCR2 as compared to LPS alone in the same samples. Similar observations have been made by others in a model of ventilator-induced ALI^15^, indicating that the chemotactic response of pulmonary cells may be suppressed in response to H_2_S administration. These results allow us to hypothesise that decreased pulmonary chemoattractant signalling due to H_2_S treatment may lead to reduced neutrophilic transmigration as we observed *in vivo*. Because our results derived from lung homogenates, we cannot differentiate between pulmonary cell types as effect sites of H_2_S. We therefore tested the cell specific effects of H_2_S on differentiated Hoxb8 neutrophils^19^ as well as in co-culture with endothelial HUVEC cells *in vitro*. In these experiments, we aimed to assess three potential mechanisms:

1. H_2_S may induce toxic effects in neutrophils, thus preventing neutrophil transmigration.
  After 24 h of incubation with the slow releasing H_2_S donor GYY4137 in concentrations ranging from 0.1 µM to 1 mM, we were not able to detect any impact on cellular vitality in Hoxb8 neutrophils. To the best of our knowledge, no other study has yet tested the effect of GYY4137 on neutrophil vitality. The concentrations used in the current study follow and even exceed GYY4137 concentrations used in comparable vitality studies in other cell lines^39,40^. It is interesting to note that GYY4137 seems to affect cell viability after 3 to 5 days at the earliest but not up to 24 h^39,40^. In this context, incubation with H_2_S enhanced short-term survival of cultured human neutrophils after 24 h in a recent study^41^. Taken together, we suggest that the observed reduction in neutrophil transmigration does not result from toxic side-effects of H_2_S.
2. H_2_S may prevent transmigration of neutrophils into the lungs.
  Upon chemoattractant signalling in the lung, neutrophils first pass the endothelial barrier and migrate to the site of inflammation^5,21^. As it has been demonstrated in a model of small intestine ischemia-reperfusion injury, H_2_S can prevent leukocyte adhesion and rolling^16–18^.
  To address our above mentioned hypothesis, we established an *in vitro* neutrophil transmigration model using an endothelial monolayer (HUVEC cells) on a perforated insert (Fig. 3b) that was co-cultured with differentiated Hoxb8 neutrophils^42^. Adding the chemoattractant cytokine MIP-2 to the bottom medium led to a substantially increased transmigration of Hoxb8 cells through the endothelial cell layer into the bottom medium as compared to the control group. Adding GYY4137 to the top medium completely inhibited this process. Our observations can be explained in several ways: First, the permeability of the endothelial barrier increased upon MIP-2 stimulation and decreased in response to H_2_S supplementation^18,43,44^, consequently reducing the capability of neutrophils to cross intercellular gaps. Second, H_2_S limits the pro-inflammatory signalling in stimulated HUVEC cells^34,45^, thus attracting fewer neutrophils. This interpretation is strengthened by our *in vivo* results in which GYY4137 inhibited MIP-2 mRNA and protein accumulation in lung tissue and BAL. While the latter two points might explain how H_2_S impacts neutrophil transmigration through the endothelium during inflammation, the present migration experiment also suggests a direct effect of GYY4137 on neutrophils. As we observed, GYY4137 significantly reduced the number of spontaneously transmigrating neutrophils even in the absence of MIP-2 stimulation.
3. H_2_S may directly inhibit neutrophil pro-inflammatory signalling and oxidative burst.

At the site of inflammation, activated and migrated neutrophils liberate pro-inflammatory cytokines and ROS in order to enhance the inflammatory process^5^. Upon LPS stimulation, we found a substantial increase of MIP-2 protein in the medium of Hoxb8 cell cultures, a response that is comparable to other neutrophil cell lines^46,47^. In contrast, we provide first evidence that additional application of GYY4137 prevented MIP-2 release, indicating that H_2_S can suppress the inflammatory response in neutrophils.

In the current study, we show that LPS stimulation directly induced ROS formation in Hoxb8 neutrophils as previously reported in isolated human neutrophils^48,49^. In contrast, H_2_S releasing GYY4137 prevented ROS production even in the presence of LPS, strongly suggesting that H_2_S profoundly inhibits the oxidative burst in neutrophils.

In conclusion, the slow-releasing H_2_S compound GYY4137 prevents lung injury and neutrophil transmigration in a mouse model of LPS-induced ALI. The inhibition of neutrophil transmigration by H_2_S appears to be a critical step for preventing lung injury. We show *in vivo* and *in vitro* that GYY4137 limits neutrophil migration by reducing chemoattractant signalling in lung tissue and endothelial cells. Moreover, we demonstrate that H_2_S directly suppresses the pro-inflammatory response and the production of ROS in neutrophils. These findings allow first insights into the cell specific effects of H_2_S and underline the beneficial potential of H_2_S releasing compounds in the prophylaxis of acute lung injury.

## Methods

### Animals

Animal experiments were performed in accordance with the guidelines of the local animal care commission (University of Freiburg, Freiburg, Germany) and in conformance with the journals’ requirements for human and animal trials (ARRIVE Animals in Research: Reporting *In Vivo* Experiments). The study was approved by the local government in consultation with an ethics committee (Regierungspräsidium Freiburg, Referat 35, Fachgebiet Tierschutz und Tierhaltung, abteilung3@rpf.bwl.de, Freiburg, Germany, permission No. G-12/73). C57BL/6 N mice (n = 32, weighing 24.6 ± 0.2 g) were obtained from Charles River Laboratories (Sulzfeld, Germany).

### Experimental setting

Preliminary experiments were conducted prior to the study in order to establish the appropriate dose for LPS-induced moderate lung injury and to determine the effective dose of intraperitoneally (i.p.) applied GYY4137 (Supplementary Fig. S1). Mice were randomly assigned into four experimental groups (n = 8/group; Fig. 1a). Group 1 (control): mice received 25 µl/g body weight phosphate buffered saline (PBS) i.p. and were exposed to synthetic air for 1 h. Afterwards, mice were treated with 5 ml of aerosolised PBS and subjected to synthetic air for another 6 h. Group 2 (control + GYY): mice received 250 mg/kg body weight GYY4137 i.p. (freshly dissolved in PBS, 10 mg/ml; Dichloromethane Complex^24^, Sigma, Taufkirchen, Germany) and were exposed to synthetic air for 1 h. Afterwards, mice were treated with 5 ml of aerosolised PBS and subjected to breathe synthetic air for another 6 h. Group 3 (LPS): mice received 25 µl/g body weight PBS i.p. and were exposed to synthetic air for 1 h. Afterwards, mice were treated with 0.05 mg of aerosolised LPS (E.coli 055:B5, Sigma^14,50^; dissolved in 5 ml PBS) and subjected to breathe synthetic air for another 6 h. Group 4 (LPS + GYY): mice received 250 mg/kg body weight GYY4137 i.p. (freshly dissolved in PBS) and were exposed to breathe synthetic air for 1 h. Afterwards, mice were treated with 0.05 mg of aerosolised LPS (dissolved 5 ml PBS) and subjected to breathe synthetic air for another 6 h. Experiments were performed in a sealed plexiglas chamber with a constant air flow of 1.5 l/min. Mice had free access to food and water. Nebulisation was performed in a custom-built cylindrical chamber (20 cm in length, 9 cm in diameter) connected to an air nebuliser (MicroAir; Omron Healthcare, Vernon Hills, IL, USA), producing particles from 1–5 μm, as previously described^21^. Nebulisation of the 5 ml solutions was ceased after 14–16 min in all experiments.

At the end of the experiment (6 h after PBS or LPS nebulisation), mice were euthanised by an overdosed injection of ketamine (180 mg/kg, i.p.) and acepromazine (1.8 mg/kg, i.p.). Bronchoalveolar lavage fluid and lung tissue for histological examination and semi-quantitative polymerase chain reaction (sq RT-PCR) analysis were gained as described previously^10,51^.

### Histological examination and ALI Score

Cryosections of the left lung lobes and haematoxylin and eosin staining were performed and analysed in a blinded fashion as described previously^10^. Alveolar wall thickness, cellular infiltration and haemorrhage were each rated from 0 (no injury) to 4 (maximal injury) for all individuals. Counts of each score were summed up, and the result was depicted as ALI score as described previously^10^.

### BAL cytokine measurements

BAL aliquots were analysed using interleukin-1β and macrophage inflammatory protein-2 ELISA kit (R&D Systems GmbH, Wiesbaden, Germany) according to the manufacturers’ instructions.

### RNA preparation and semi-quantitative polymerase chain reaction (sqRT-PCR)

RNA from lung tissue samples was extracted and purified as previously described^51^. cDNA samples were synthesised from equal amounts of RNA using random hexamer reverse primers and a TaqMan Reverse Transcription kit (Applied Biosystems Inc., Foster City, USA). TaqMan PCR reactions were performed according to the manufacturers’ instructions. TaqMan Gene Expression Assays for CXCL2 (MIP-2; Mm00436450_m1), CXCR2 (Mm00438258_m1), and GAPDH (TaqMan Rodent GAPDH Control Reagent) were purchased from Applied Biosystems. The comparative CT (ΔΔCT) method to evaluate the expression profiles of the analysed samples was used.

### Cell culture

Human umbilical vein endothelial cells (HUVEC, Pelobiotech, Planegg, Germany) were cultured in complete endothelial cell growth medium (Promocell GmbH, Heidelberg, Germany) supplemented with 10% fetal calf serum (FBS, Gibco, Life Technologies GmbH, Darmstadt, Germany) and 1% penicillin-streptomycin (Promocell). Hematopoietic progenitor Hoxb8 neutrophils^19^ were a generous gift from Prof. Häcker (Institute of Medical Microbiology and Hygiene, UMC, Freiburg, Germany). Cells were cultured in Opti-modified Eagle medium (Gibco) supplemented with 10% heat-inactivated FBS (Gibco), 30 µM 2-mercaptoethanol (Thermo Fischer, Paisley, UK), 1 µM estradiol (Sigma) and 1% stem cell factor. Stem cell factor was harvested from chinese hamster ovary cells (a generous gift from Prof. Häcker) as described earlier^19^. Prior to the experiments, progenitor Hoxb8 neutrophils were cultured for four days in differentiating medium (estradiol free medium)^19^. All cells were grown and experiments were performed at standard growing conditions (37 °C, 5% CO_2_, sufficient humidity). All neutrophil assays were performed in the presence of FBS.

### Neutrophil vitality testing

10^6^/3 ml differentiated neutrophils were seeded in 6-well plates. Prior to the onset of incubation, 300 µl of the cell suspension were removed, stained for propidium iodide (PI, Thermo Fisher, Waltham, USA), and analysed by fluorescence-activated cell sorting (FACS, Invitrogen™ Attune™, Thermo Fisher). Remaining cells were incubated in the absence (control) or presence of GYY4137 (GYY) for 24 h. Different concentrations of GYY4137 were used: 1 mM, 0.1 mM, 10 µM, and 1 µM. At the end of the experiment, cells were harvested, stained for PI, and analysed by FACS. The relative amount of vital cells was calculated as the ratio between vital cell counts at the beginning and the end of the experiment. For subsequent short-term experiments, a dose of 1 mM GYY4137 was chosen to achieve appropriate effects without toxic side-effects^31,52,53^.

### Neutrophil transmigration assay

HUVEC were seeded onto cell culture inserts (pore size 3.0 µm) and grown to confluence. Prior to experiments, cells were washed and the inserts were transferred to new 6-well companion plates. The lower compartment of each well was filled with 3 ml of neutrophil cell culture medium. The upper compartments were filled with 1 ml of 5 × 10^6^ differentiated neutrophils (experimental design, Fig. 3b). As controls, the co-culture setup was performed in the absence (control) or presence of 1 mM GYY4137 (control + GYY). The latter was added to the upper compartment. For stimulation of transmigration, MIP-2 (1.33 pg/ml) was supplemented to the lower compartment in the absence (MIP-2) or presence of 1 mM GYY4137 (MIP-2 + GYY). Inserts were removed from the companion plates after 2 h of incubation. Cells from the lower and upper compartment were stained separately with PI (3.75 µM) and subsequently the live/dead cell ratio was analysed by FACS.

### Neutrophil cytokine measurement

Differentiated neutrophils were seeded in 24-well plates. As controls, cells were incubated in the absence (control) or presence of 1 mM GYY4137 (control + GYY) for 4 h. In addition, cells were incubated with 100 ng/ml LPS in the absence (LPS) or presence of 1 mM GYY4137 (LPS + GYY) for 4 h. Neutrophil cell culture supernatants were analysed using MIP-2 ELISA kit (R&D Systems GmbH) according to the manufacturers’ instructions.

### Neutrophil detection of reactive oxygen species

Differentiated neutrophils were seeded in 24-well plates. As controls, cells were incubated in the absence (control) or presence of 1 mM GYY4137 (control + GYY) for 4 h. In addition, cells were incubated with 100 ng/ml LPS in the absence (LPS) or presence of 1 mM GYY 4137 (LPS + GYY) for 4 h. Subsequently, neutrophils were stained with 2′,7′-dichlorodihydrofluorescein diacetate (DHDHF-DA, Sigma) in order to detect reactive oxygen species as previously described^54^. Fluorescence was measured with TECAN infinite 2000 (Thermo Fisher).

### Statistical analysis

*In vivo* experiments were performed with n = 8 mice per group. Power calculations were performed prior to the study in order to define group sizes. Cell culture experiments were performed from at least three subsequent cell passages with n = 3 per group. Graphs represent means ± standard error of means (SEM) and were created with SigmaPlot 11.0 software (Systat Software Inc., Erkrath, Germany). In Figs 2a,b and 4b, data were depicted as fold induction compared to untreated controls. Data were further analysed for normal variation prior to one way analysis of variance (ANOVA) followed by the Tukey’s post hoc test. P < 0.05 was considered significant. All calculations were performed with GraphPad Prism 7.01 (GraphPad Software, Inc., La Jolla, CA, USA).

## Electronic supplementary material

Supplementary Figure S1

## Data Availability

All data generated or analysed during this study are included in this published article (and its Supplementary Information file).
