# Supplementary material for: Hydrogen sulfide limits neutrophil transmigration, inflammation, and oxidative burst in lipopolysaccharide-induced acute lung injury
Source: Sci Rep. 2018 Oct 2;8:14676. doi: 10.1038/s41598-018-33101-x (PMC6168479; doi:10.1038/s41598-018-33101-x)
Supplement: Supplementary file 1 — Supplementary Figure S1 [file 41598_2018_33101_MOESM1_ESM.pdf]

Hydrogen sulfide limits neutrophil transmigration, inflammation, and oxidative burst in lipopolysaccharide-induced acute lung injury

Simone Faller[+]*, Florian Hausler[+], Andreas Goeft, Marc-Nicolas André von Itter, Veronica Gyllenram, Alexander Hoetzel, and Sashko G Spassov

Department of Anesthesiology and Critical Care Medicine, Medical Center – University of Freiburg, Faculty of Medicine, University of Freiburg, Germany

*corresponding author:
simone.faller@uniklinik-freiburg.de

[+]both authors contributed equally to the manuscript

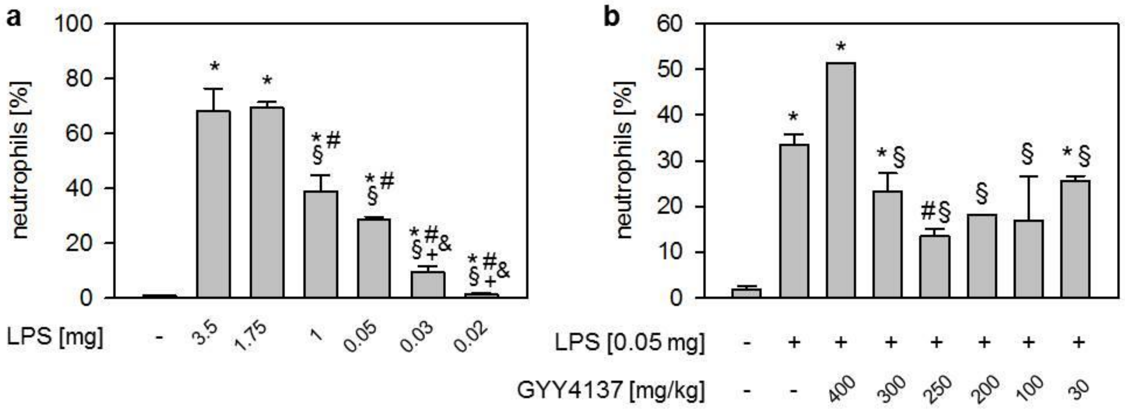

Supplementary Figure S1.
Effect of varying LPS and GYY4137 doses on neutrophil influx into the BAL.
(a) Mice were nebulised with LPS (dissolved in 5 ml PBS) in concentrations ranging from 3.5 to 0 mg. All mice were euthanised after another 6 h. The relative amount of neutrophils in the bronchoalveolar lavage was determined by cytospin analysis. Data represent means ± SEM for n=2-6/group. ANOVA (Tukey´s post hoc test), *P<0.05 vs. control; #P<0.05 vs. LPS 3.5 mg; §P<0.05 vs. LPS 1.75 mg; +P<0.05 vs. LPS 0.1 mg; &P<0.05 vs. LPS 0.05 mg.
(b) Control mice received 25 µl/g PBS i.p. and were nebulised 1 h later with 5 ml PBS. The LPS treated mice received 25 µl/g PBS i.p. and were nebulised 1 h later with 0.05 mg LPS (dissolved in 5 ml PBS), while the LPS+GYY groups were treated with 400, 300, 250, 200, 100 and 30 mg/kg GYY4137 i.p. as indicated and were nebulised 1 h later with 0.05 mg LPS. All mice were euthanised 6 h after LPS treatment. The relative amount of neutrophils in the bronchoalveolar lavage was determined by cytospin analysis. Data represent means ± SEM for n=1-4/group. ANOVA (Tukey´s post hoc test), *P<0.05 vs. control; #P<0.05 vs. LPS; §P<0.05 vs. LPS+400mg/kg GYY.
